# Ablation of Intracavitary Structures: Anatomy, Anatomy, Anatomy

**DOI:** 10.19102/icrm.2018.090206

**Published:** 2018-02-15

**Authors:** Jason S. Bradfield, Kalyanam Shivkumar

**Affiliations:** ^1^UCLA Cardiac Arrhythmia Center, David Geffen School of Medicine at UCLA, Los Angeles, CA, USA

**Keywords:** Anatomy, intracavitary, moderator band, papillary muscle

In this issue of *The Journal of Innovations in Cardiac Rhythm Management*, Donnelly et al. provide a comprehensive review of ventricular arrhythmias arising from the left ventricular papillary muscles and the techniques used for mapping and ablation.^1^

When premature ventricular contraction or ventricular tachycardia ablation fails from an endocardial approach, patients are often sent to specialized centers for consideration for epicardial mapping and ablation. Yet, in many instances, the site of origin of the ventricular arrhythmia is as far away from the epicardium as is anatomically possible: the intracavitary structures, specifically the papillary muscles.

As a field, to both enhance our individual skills and improve procedural success rates, we must return to the basics. Without understanding the beauty and complexity of cardiac anatomy, procedural skills can only reach a certain threshold. For instance, many expect the posteromedial papillary muscle to be septal. However, as can be seen in **Figure 1**, both papillary muscles are predominantly lateral structures. Further, the moderator band in the right ventricle is a frequent site of ventricular arrhythmia origin and has highly variable size and right ventricular free wall insertion sites.

An in-depth understanding of anatomy doesn’t preclude the need to have technical skill, but, without adequate anatomic knowledge, even the most technically skilled operators will still be at risk for a high rate of procedural failure.

Technical failures are sometimes unavoidable, but every effort should be made to ensure cognitive failures do not occur.

## Figures and Tables

**Figure 1:**
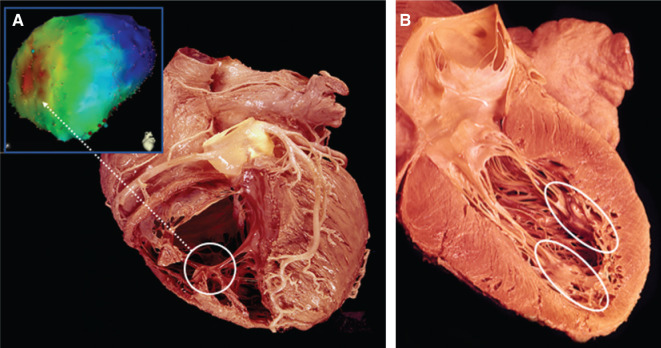
Anatomic images of the heart in the left anterior oblique **(A)** and right anterior oblique views **(B)**. The more lateral location of both the anterolateral and posteromedial left ventricular papillary muscles are shown in **B** (white ovals). The insertion of the right ventricular moderator band to the right ventricular free wall is shown in **A**. The inset in **A** shows a three-dimensional electroanatomic map (CARTO™; Biosense Webster, Diamond Bar, CA, USA) of the epicardial sinus rhythm activation. This map demonstrates the earliest site of epicardial activation on the right ventricular free wall, due to rapid moderator band conduction to this region. Image courtesy of the Wallace A. McAlpine UCLA Collection.
